# A Combination Of Vein Of Galen Aneurysmal Malformation And Bovine Aortic Arch In Newborn: A Case Report

**DOI:** 10.31729/jnma.8987

**Published:** 2025-05-31

**Authors:** Krishna Deo Mandal, Kalpana Subedi, Unnati Amatya, Janak Pathak

**Affiliations:** 1 Department of Pediatrics and Neonatology, Paropakar Maternity and Women's Hospital, Thapathali, Kathmandu, Nepal; 2 Obstetrics and Gynecology, Embassy of India, Kathmandu, Nepal

**Keywords:** *arteriovenous malformation*, *bovine aortic arch*, *congenital anomalies*, *vein of Galen malformation*

## Abstract

Vein of Galen aneurysmal malformation is a rare intracranial arteriovenous malformation, coexistence with aortic arch anomalies is even more uncommon, carry high risk of morbidity and mortality related to endovascular procedure and poor neurological outcome. Vein of Galen aneurysmal malformation is a dilated venous pouch, a persistence of the embryonic median prosencephalic vein of Markowski. Often presents with high output cardiac failure, severe pulmonary hypertension, and systemic steal leading to hemodynamic compromise and multi organ failure. Bicetre score scale determines the potential treatment option and prognosis of the disease. Aim of the medical treatment is initial stabilization of life threatening condition of severe heart failure followed by the definitive treatment with endovascular embolization. Treatment is a big challenge, specially if coexistence with aortic arch anomalies which increases the morbidity and mortality. We describe a term neonate found to have vein of Galen aneurysmal malformation associated with bovine aortic arch.

## INTRODUCTION

Vein of Galen results from the persistence of the embryonic median prosencephalic vein (MPV) of Markowski, which is normally replaced by the internal cerebral veins during neural vascularization.^1^ Dilatation of the vein due to cerebral arteriovenous malformation leads to Vein of Galen aneurysmal malformation (VGAM).^1,2^ It accounts for 1% of pediatric intracranial AV malformations and occurs in fewer than 1 in 25,000 births.^1,2^ At birth, it may present with high- output cardiac failure, pulmonary hypertension, and systemic steal, causing hemodynamic compromise.^2-4^ VGAM with anomalous aortic arch is rare and associated with higher mortality and poor outcomes.^5-7^ Bovine aortic arch, the most common anomaly, has a prevalence of 7-25%.^6-7^ We report a term neonate with coexisting VGAM and bovine aortic arch.

## CASE REPORT

**Figure 1:**
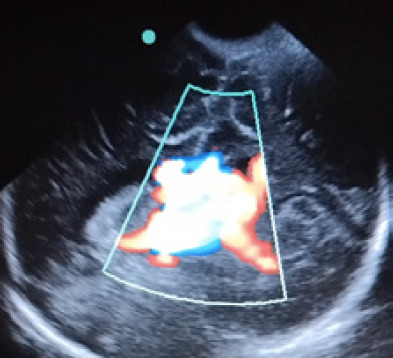
Cranium ultrasound showing dilated intracranial vessels with increased flow.

A term female was born via elective cesarean section to a 30 years old primigravida following an uncomplicated pregnancy with Apgar score 7/10 and 9/10, birth weight of 2900 grams. Her antenatal ultrasound finding were normal, but she dint have fetal echocardiography. Soon after birth, she developed respiratory distress and became cyanosed needing supplemental oxygen via continuous positive airway pressure(CPAP) support. Congenital cardiac anomaly was suspected in view of cardiac murmur. Arterial blood gas, complete blood count, electrolytes and inflammatory marker were within normal range. Chest X-ray showed mild cardiomegaly. Echocardiography of heart showed patent ductus arteriosus (PDA) and foramen ovale (PFO) both bidirectional shunt, severe tricuspid valve regurgitation (TR), TR pressure gradient; 50 mmHg, suggestive of severe pulmonary hypertension (PAH). In addition there was abnormal branching pattern of aortic arch with presence of bovine aortic (Figure 1).

There was diastolic flow reversal seen in aorta with increased blood flow in superior vena cava (SVC). Further CT angiography of heart confirmed left side type A bovine aortic arch (BBA). The patient was started on sildenafil for severe PAH, maintenance intravenous (IV) fluid along with IV antibiotics. Later IV milrinone infusion was added. The presence of retrograde flow in descending aorta gave a clue for low resistance cerebral arteriovenous malformation leading to the diastolic steal phenomenon. Auscultation of anterior fontanel had bruit which suggested possible intracranial AV malformation. Bedside head ultrasound showed dilated intracranial vessels with increased vascularity and blood flow, (Figure 1).

**Figure 2:**
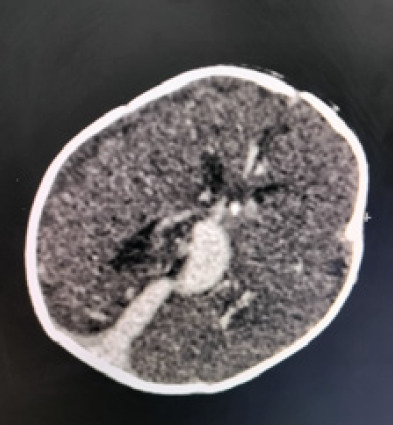
Contrast enhanced CT of head showing vein of Galen aneurysmal dilations with multiple dilated feeding arteries.

Contrast-enhanced CT of head confirmed the diagnosis of vein of Galen aneurysmal malformation, (Figure 2).

Her Bicetre score was calculated to be between 12 and 14. Neuro consultation was done who advised to continue medical management and to monitor Bicetre score closely and planned for the endovascular embolization if Bicetre score became less than 12 on close follow-up or at 6 months of life. On 12^th^ days of life patient had increasing respiratory distress, generalized edema, and hepatomegaly due to congestive cardiac failure. The baby continued on CPAP. IV fluid restriction was done and IV Frusemide infusion was started. Lab test showed deranged liver enzymes and coagulation profile which was treated with vitamin K and fresh frozen plasma. Parents were counseled about the treatment option of VGAM and in addition the prognosis when its association with BAA were discussed. On 18^th^ days of life the patient was discharged on parents' request due to financial constraint and poor prognosis on oral diuretic and nutritional supplement and her parents were traced through phone call, the baby expired on 49th day of life. Outcome remains uncertain.

## DISCUSSION

Persistence of the embryonic MPV of markowski, which is the first vein to drain the choroid plexuses of the lateral and third ventricles between the 7^th^ and 12^th^ weeks of gestation and replaced by the internal cerebral veins, except most caudal portion of MPV which form the vein of Galen.^1-3^ VGAM consists of multiple arteriovenous shunt draining into a dilated vein of Galen.^1^ The most common feeders are the posterior choriodal arteries followed by anterior cerebral artery.^1,2^ There are two types of VGAM.^4^ The choroidal type, is a primitive condition, most predominant and encountered in neonatal period with severe form of cardiorespiratory symptoms. similar to the present case, VGAM was the choroidal type and symptoms started soon after birth. In contrast to that, the mural type, direct arteriovenous fistula within the wall of the MPV, often is better tolerated and encountered in infancy with intracranial hemorrhage, headache, seizure, neurological deficits and hydrocephalus.^1-3^ Ultrasound and MRI are the essential tools for diagnosis of VGAM in utero, prognostic evaluation and optimal management of such pregnancy.^2^ During fetal life VGAM well tolerated due to a low resistance placental circulation. However, after birth the flow through the VGAM increases significantly leading to high output cardiac failure, pulmonary hypertension, neurological, hepatic and renal dysfunction^1-3^ similar to the present case. Up to 80% of cardiac out put is directed toward the VGAM, which leads to increased venous return to the right side of the heart leading to heart failure and persistence of right to left shunt through PFO and PDA and subsequent PAH. In addition, circulatory steal to the VGAM produce the characteristic diastolic flow reversal in the descending aorta and potentially multi organ failure.^1-4^ We have similar finding in our case except for renal failure and neurological dysfunction.

According to Lasjaunias et.al use of Bicetre score scale based on cardiac, respiratory, cerebral, renal and liver function to determine potential treatment option. A total 21-points scale gives the severity of the disease. Bicetre score less than 8 suggestive of poor prognosis, score 8 to 12 needs emergency endovascular embolization, and score more than 12 is indication of medical managements until five months of age.^4^ Our patient had Bicetre score in between 12 and 14, medical management was initiated. The main aim of treatment is immediate stabilization of life-threatening condition. Severe heart failure is managed by providing intensive care support, inotropes, vasodilator and diuretics to maintain hemodynamically stability prior to endovascular treatment. If refractory to medical management patient must under go emergency endovascular embolization.^2,8,9^ Currently, stage trans-arterial embolization of VGAM is the treatment of choice with favorable outcome,^2-4,8^ and prevention of severe brain damage.^2^ Despite treatment, the primary morbidity is hydrocephalus which leads to intellectual disability and high mortality up to 63% and also needs CSF shunting.^2,4,9^ Present case had VGAM coexist with BAA, an abnormal branching pattern of the aortic arch, a left carotid artery arises from brachiocephalic trunk instead of left aortic wall.^6^ Type A BAA has the common origin of left carotid artery and brachiocephalic trunk, whereas type B BAA, the left carotid artery originates directly from brachiocephalic trunk.^6^ Patient with this anomaly increase risk of morbidity like infarction, hemorrhage, and vascular injury related to endovascular and surgical procedure.^5,6,7^ A multicenter study showed BAA is the independent risk factor for poor neurological outcomes and has direct impact on mortality.^6,7,10^ Keeping in mind, all these factor constitute poor outcome in the patient with BAA and VGAM, an unfavorable combined anatomic variation was present in our case. Prenatal screening for these anomalies, can help diagnose this case in-utero and postnatal avoidance of such unfavorable condition.

## CONCLUSION

VGAM is a rare intracranial AV malformation and Coexistence with aortic arch anomalies is even more uncommon. The coexistence of these two condition carries high risk of morbidity and mortality related to endovascular procedure, and poor neurological outcome. This condition must be ruled out prenatally with improvement in antenatal fetal anomaly screening and fetal echocardiography. Initial treatment is to relieve the congestive heart failure, circulatory stabilization until the definitive treatment with endovascular procedure is done. Despite treatment, VGAM coexistence with other cardiac anomalies carry worse prognosis.
